# A benzimidazole inhibitor attenuates sterile inflammation induced in a model of systemic autoinflammation in female mice

**DOI:** 10.1038/s41598-020-68985-1

**Published:** 2020-07-21

**Authors:** Federica Agliano, Keaton S. Karlinsey, Michael Ragazzi, Antoine Ménoret, Anthony T. Vella

**Affiliations:** 1 Department of Immunology, University of Connecticut Health Center, Farmington, CT USA; 2 Institute for Systems Genomics, UConn Health, Farmington, CT USA

**Keywords:** Immunology, Diseases

## Abstract

Sterile stimuli can trigger inflammatory responses, and in some cases can lead to a variety of acute or chronic diseases. In this study, we hypothesize that a benzimidazole inhibitor may be used as a therapeutic in the treatment of sterile inflammation. In vitro, this inhibitor blocks TLR signalling and inflammatory responses. The benzimidazole inhibitor does not prevent mouse macrophage activation after stimulation with 2,6,10,14-tetramethylpentadecane (TMPD, also known as pristane), a hydrocarbon oil that mimics features of sterile inflammation when injected in vivo. However, C57BL/6J female mice treated with the benzimidazole inhibitor exhibited a significant reduction of pristane-dependent induction of splenocyte number and weight. Conversely, no significant difference was observed in males. Using mass spectrometry, we found that the urine of pristane-injected mice contained increased levels of putative markers for several inflammatory diseases, which were reduced by the benzimidazole inhibitor. To study the mechanism, we showed that pristane-injected mice had increased cell free DNA in serum, which was not impacted by inhibitor treatment. However, chemokine release (e.g. MCP-1, RANTES and TARC) was significantly reduced in inhibitor-treated mice. Thus, the benzimidazole inhibitor might be used as a new drug to block the recruitment of immune cells during sterile inflammatory diseases in humans.

## Introduction

Sterile inflammation is defined as an inflammatory condition triggered by sterile stimuli, such as toxins, minerals and chemicals^1,2^, rather than proinflammatory molecules belonging to pathogenic microbes^3^. Similar to pathogen-associated inflammation, sterile inflammation can be initiated by activation of Pattern Recognition Receptors (PRRs), including Toll-like Receptors (TLRs), and leads to production of proinflammatory mediators^1^. Furthermore, failure to promptly remove or contain agents causing sterile inflammation can be harmful to the host, leading to chronic inflammation. Nevertheless, it is also possible that sterile inflammation opens space for infection or for the microbiome to exacerbate inflammatory events. Examples of sterile inflammatory disorders are ischemia–reperfusion injury^4^, arteriosclerosis^5^, Alzheimer’s disease^6^, and other autoinflammatory and autoimmune diseases^7^. Although in the last few years several compounds, such as oridonin^8^ and CY-09^9^ have been shown to elicit potent therapeutic effects in mouse models of inflammatory diseases; the discovery of new possible anti-inflammatory drugs may lead to the development of more effective therapies towards sterile inflammatory diseases.

Here, we used a model of sterile inflammation where the triggering agent is 2,6,10,14-tetramethylpentadecane (TMPD, also known as pristane). Pristane is a naturally occurring hydrocarbon oil found in small quantities in many plants, in various marine organisms, and as the most active component of mineral oil^10^. Importantly, there is evidence that properties of certain hydrocarbons such as pristane can mediate inflammatory or autoimmune disease in humans and animals^11–13^. Furthermore, pristane is well-known to induce some features of chronic inflammation when introduced into the peritoneal cavity in mice^14,15^. A number of reports have shown adaptive immune responses towards pristane, especially in the context of a Lupus-like disease^14,16–18^; nevertheless there are still outstanding questions on whether pristane can also affect innate immune responses, an area that should be explored to comprehensively understand therapeutic intervention on sterile inflammatory diseases.

In order to block inflammation in vitro and in vivo, we used a benzimidazole inhibitor. This inhibitor was previously shown to have a major effect in inhibiting IRAK1 and IRAK4^19^, two serine/threonine kinases involved in a full spectrum of TLR/IL-1R responses^20^. Hence, in this paper we demonstrate that treatment with a benzimidazole inhibitor results in a significant reduction of TLR-dependent inflammatory response. Furthermore, mouse macrophages stimulated with pristane exhibit signs of activation, such as increased cell size and RANTES production. The benzimidazole inhibitor reduces pristane-induced splenomegaly and chronic inflammatory urine biomarkers in C57BL/6J females. But interestingly pristane induces an increase in serum DNA even after inhibitor treatment, whereas serum proinflammatory chemokines were significantly reduced in inhibitor-treated mice. The purpose of this study was to investigate whether and how the benzimidazole inhibitor could reduce inflammation in vitro and in vivo*,* using a model of systemic autoinflammation. Our results suggest that the use of the benzimidazole inhibitor as a therapeutic should allow amelioration of innate immune responses to sterile inflammatory diseases, mainly targeting IRAK1 and IRAK4.

## Methods

### Mice and treatment

C57BL/6J mice were obtained from the Jackson Laboratory (Bar Harbor, ME). Mice were housed in the University of Connecticut Health Center animal facility, and 6–12 week-old male and female mice were used. All animal procedures were approved by the UConn Health Institutional Animal Care and Use Committee and performed in accordance with National Institutes of Health Animal Care and Use Guidelines. In order to induce inflammation in mice, the animals were intraperitoneally (i.p.) injected with 0.5 ml of pristane (2,6,10,14-tetramethylpentadecane (TMPD)) (Sigma, St. Louis, MO) as previously described^21–24^. PBS was administered to the control group. After 7 days, mice received an i.p. injection twice per week of either benzimidazole inhibitor (I5409, Sigma, St. Louis, MO; also known as 1-(2-(4-Morpholinyl)ethyl)-2-(3-nitrobenzoylamino)benzimidazole) (60 µg/mouse) or vehicle (DMSO) as previously described^25^.

### Generation and stimulation of bone marrow-derived macrophages

To generate bone marrow-derived macrophages (BMDMs), bone marrow cells from mouse femurs and tibias were differentiated for 7 days in DMEM (Gibco, Dublin, Ireland), supplemented with 10% FBS (Atlanta Biologicals, Flowery Branch, GA), 2 mM l-glutamine, 100 U/ml penicillin, 100 μg/ml streptomycin, HEPES buffer and 15% L929 cell-conditioned, macrophage-colony-stimulating factor-containing supernatant^26^. BMDMs were pre-treated with increasing concentrations of the benzimidazole inhibitor (0 to 10 μM). After 30 min cells were stimulated with increasing concentrations of CpG (ODN 2395, Innaxon, United Kingdom), *Salmonella enterica*-derived LPS (Sigma, St. Louis, MO), or Poly I:C (Sigma, St. Louis, MO) for 18 h, with the inhibitor still present during the entire stimulation period. Where indicated, pristane was added as an inclusion complex with β-cyclodextrine (β-CD) (Sigma-Aldrich) as previously described^27^.

### Mouse urinary proteomics analysis

Each urine sample was analysed at the UCONN Proteomics & Metabolomics Facility*.* Samples were subject to Cys reduction and proteolysis was achieved using sequencing grade trypsin (Promega). Equimolar peptide aliquots were separated using a 60 min nanoflow ultra-high performance liquid chromatography (UPLC) reversed-phase gradient on an Ultimate 3,000 RSLCnano UPLC instrument (Thermo Scientific). Eluted peptides were ionised directly into a Thermo Scientific Q Exactive HF hybrid quadrupole-Orbitrap instrument implementing high resolution tandem mass spectrometry (MS/MS) and electrospray ionization (ESI) with the following parameters: positive ESI mode, 60 K and 15 K resolution for MS and MS/MS scans, respectively, MS mass range 300 to 1,800 m/z, Top 15 data-dependent MS/MS acquisition. Peptide/protein identification and label-free quantitation (LFQ) was achieved by searching against the Uniprot *Mus musculus* proteome database (accessed 2017 May 16) using the Andromeda and MaxQuant software package (v1.6.0.1)^28^ and the following parameters: 1% false discovery rate cutoff, trypsin cleavage specificity with up to 2 missed cleavages, variable modifications: oxidised Met, N-terminal acetylation, deamidation of Asn/Gln, peptide N-terminal Gln to pyroGlu, fixed carbamidomethylation on Cys, “LFQ” protein quantification active, and a minimum of 5 amino acids/peptide. All other parameters were assigned default values. Search results were uploaded into Scaffold Q + S (v4.9.0, Proteome Science) for visualization and further analysis. Refer to “Data availability” section for more information on raw data.

### Cell-free (Cf) DNA isolation and quantification

DNA was extracted from 100 μl serum using DNA Extractor SP Kit (FUJIFILM Wako Chemicals U.S.A. Corp.), according to the manufacturer’s instructions. Next, cell-free DNA was quantified using the Quant-iT PicoGreen dsDNA Assay Kit (Invitrogen) according to the manufacturer’s instructions.

### Flow cytometry

After stimulation, cells were detached, washed in wash buffer and run in a BD LSR II flow cytometer. Cells were gated in a SSC-A**/**FCS-A plot**.** To exclude doublets and/or clumps, single cells were gated in a FCS-H**/**FSC-A plot. Subsequently, using the FSC-A parameter, cell sizes were compared. Data were analysed using FlowJo software (Tree Star Inc., Ashland, OR, USA).

### Cytokine secretion analysis

Supernatants from cell cultures were cleared by centrifugation (2,000 g, 10 min). Levels of RANTES and IL-6, known to be associated with a variety of inflammatory disorders, including sterile inflammatory diseases, were used as experimental read-out^29,30^ and determined by ELISA using commercial kits: Ccl5/Rantes (R&D Systems), IL-6 (BioLegend, San Diego, CA). Cytokine and chemokine levels in serum were measured using LEGENDplex multi-analyte flow assay kits (BioLegend, San Diego, CA) as per manufacturer’s instructions. Data were collected on MACSQuant Analyzer 10 (Miltenyi Biotech, Germany) and analysed using LEGENDplex Data Analysis V8 software (BioLegend).

### Western Blot analysis

In order to obtain whole cell lysate, cells were lysed in lysis buffer (50 mM Tris–HCl, pH 7.4, 150 mM NaCl, 1% Triton X-100, 1 mM EDTA, 5 mM NaF, 2 mM sodium orthovanadate, 1 mM PMSF, 1X complete protease inhibitors). To evaluate p65 translocation, cells were lysed with NE-PER Nuclear and Cytoplasmic Extraction Reagent Kit (Thermo Scientific) for nuclear and cytoplasmic extractions, according to the manufacturer’s instructions. Lysates were resuspended in Laemmli buffer (50 mm Tris–Cl, pH 6.8, 10% glycerol, 2% SDS, 0.1% bromophenol blue, 5% 2‐mercaptoethanol), boiled for 5 min, separated on 4–20% polyacrylamide gels (Bio-Rad), transferred to nitrocellulose membranes (Bio‐Rad), blocked, and probed with the following antibodies: ERK (# 9102)^31^, p-ERK (# 9101)^32^, p65 (# 3034)^33^, β-tubulin (# 2146)^34^ and HDAC1 (# 5356)^35^ (Cell Signaling), and developed by chemiluminescence (Thermo Fisher Scientific, Waltham, MA, USA).

### Statistical analysis

Data were processed by the GraphPad Prism Version 8.0 software package (GraphPad Software, San Diego, CA), using one-way ANOVA with Bonferroni's post-hoc test or two-way ANOVA with Dunnett’s post-hoc test to compare multiple experimental groups, or using Student's *t-*test to perform pair-wise comparisons. Data were expressed as mean ± SEM. Values of *p* < 0.05 (*) were used as significant threshold; *p* < 0.01 is indicated as (**) and *p* < 0.001 as (***).

## Results

### The benzimidazole inhibition reduces release of proinflammatory mediators and signalling in BMDMs stimulated with TLR agonists

**Figure 1:**
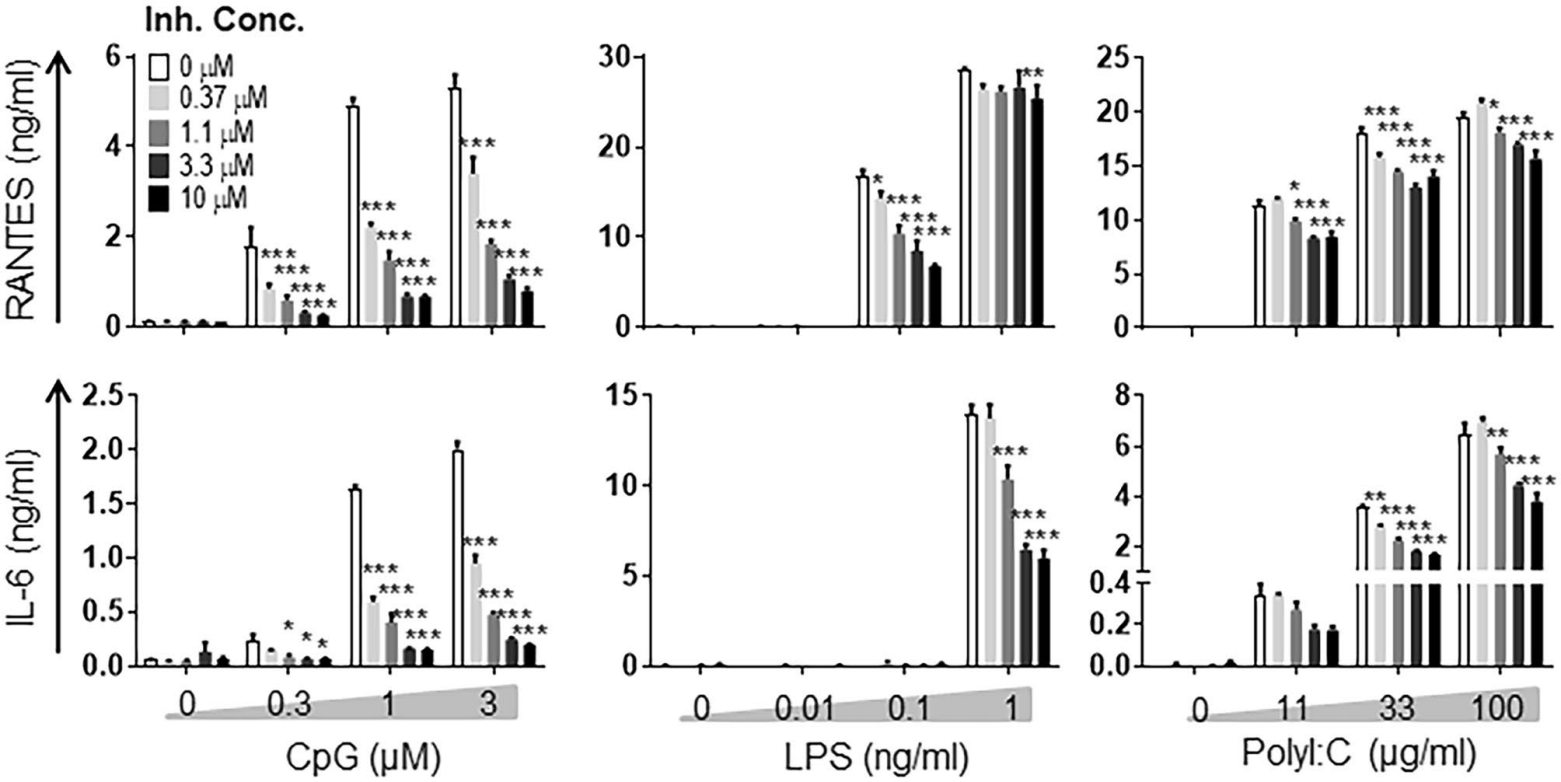
The benzimidazole inhibitor reduces proinflammatory cytokine/chemokine release in BMDMs stimulated with TLRs agonists. BMDMs were pre-treated with increasing concentrations of the benzimidazole inhibitor (0 to 10 μM). After 30 min cells were stimulated with increasing concentrations of CpG, LPS or Poly I:C for 18 h. The secretion of RANTES and IL-6 was measured by ELISA. A two-way ANOVA with Dunnett’s post-hoc test compared to the inhibitor untreated cells was performed to determine statistical significance. Representative data from 3 independent experiments (n = 3 mice per condition) are shown. *p < 0.05; **p < 0.01; ***p < 0.001.

**Figure 2:**
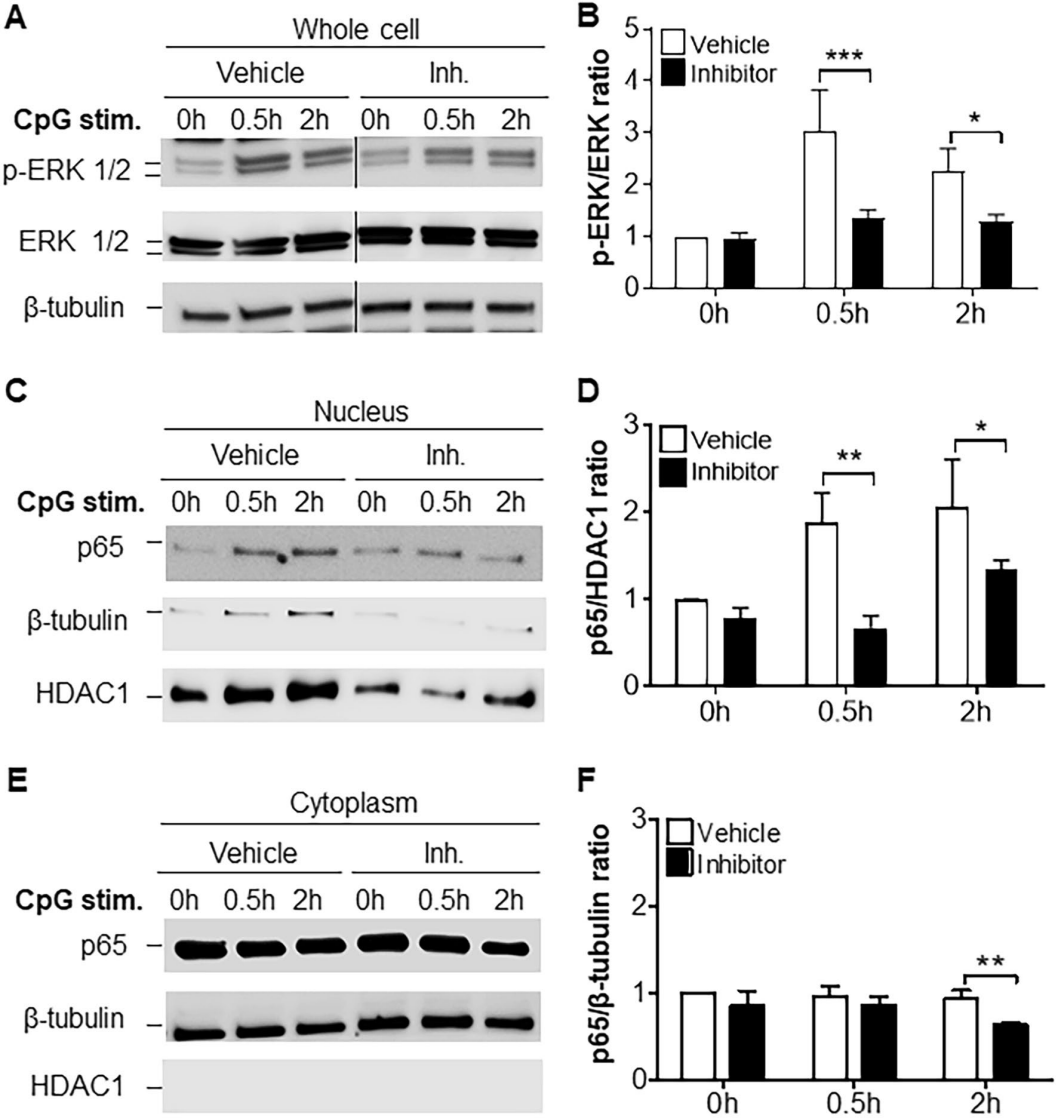
The benzimidazole inhibitor reduces TLR9 signaling in BMDMs stimulated with CpG. Cells were pre-treated with either vehicle or the benzimidazole inhibitor (10 μM) for 30 min and stimulated with CpG at different time points. (**A**) Western Blot of ERK 1/2 and p-ERK 1/2 expression in whole lysates of BMDMs. (**B**) Quantification of ERK 1/2 phosphorylation showed in (**A**). (**C**) Western Blot of nuclear p65 pattern in BMDMs. (**D**) Quantification of nuclear p65 showed in (**C**). (**E**) Western Blot of cytoplasmic p65 pattern in BMDMs. (**F**) Quantification of cytoplasmic p65 showed in (**E**). A two-way ANOVA with Bonferroni’s post-hoc test was performed to determine statistical significance. (**A**), (**C**) and (**E**) show a representative blot out of 3 independent experiments. (**B**), (**D**) and (**F**) show data from 3 biological replicates (n = 3 mice per condition) (mean ± SEM). *p < 0.05; **p < 0.01. Full-length blots are presented in Supplementary Figure S1.

The benzimidazole inhibitor was discovered to bind the kinase domain of both IRAK1 and IRAK4, with an inhibitory concentration (IC_50_) of 300 nM and 200 nM, respectively^19^. Here, we confirm the inhibitor efficiency on BMDMs stimulated with classical TLR agonists: CpG (TLR9), LPS (TLR4) and Poly I:C (TLR3). Compared to control cells, CpG stimulated BMDMs that previously received different doses of the benzimidazole inhibitor produced significantly lower levels of proinflammatory mediators RANTES and IL-6 in an inhibitor dose-dependent manner (Fig. 1, left panel). Conversely, in LPS-stimulated cells, the benzimidazole inhibitor could significantly reduce only IL-6 release but not RANTES at the higher dose of LPS (Fig. 1, middle panel). Furthermore, we found that the benzimidazole inhibitor reduced RANTES and IL-6 release in BMDMs stimulated with the TLR3 (which engages in TRIF-dependent signalling) agonist Poly I:C (Fig. 1, right panel). We further examined the effects of the benzimidazole inhibition on signalling molecules downstream of TLR9 activation. BMDMs pre-treated with the benzimidazole inhibitor or vehicle, followed by stimulation with CpG for different time points showed a significant reduction of ERK-MAP kinase phosphorylation compared to control cells after 0.5 and 2 h (Fig. 2A,B and Supplementary Fig. S1A). Furthermore, we demonstrated that the benzimidazole inhibition significantly reduced NFκB nuclear translocation (Fig. 2C,D and Supplementary Fig. S1B), having a minor effect on cytoplasmic NFκB levels (Fig. 2E,F and Supplementary Fig. S1B). Collectively these results demonstrate that the benzimidazole inhibitor efficiently blocks TLR-dependent pathways in vitro in a dose-dependent manner, and this inhibition is critical for the regulation of TLR-dependent downstream events including proinflammatory mediator production in mouse macrophages.

### The benzimidazole inhibitor reduces systemic chronic inflammation including biomarkers, but is not able to decrease serum cell-free (cf) DNA release

**Figure 3:**
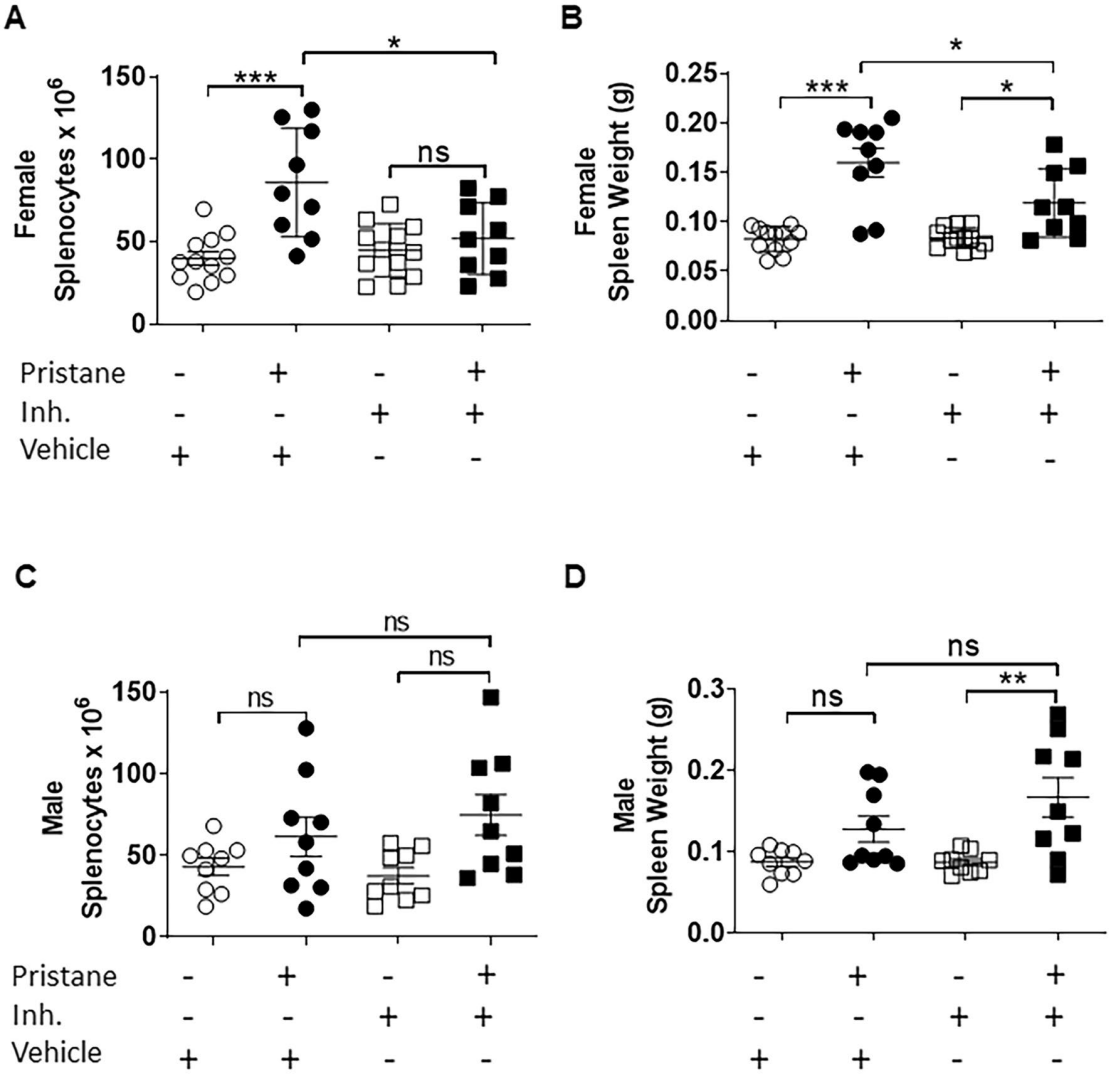
IRAK1/4 inhibitor reduces immune cell proliferation and infiltration only in spleen of female mice injected with pristane. WT male and female mice received a pristane injection (0.5 ml) and after one week were treated with DMSO or IRAK 1/4 inhibitor twice a week for 8 weeks. (**A**) Splenocyte number from each group in female mice. (**B**) Spleen weight from each group in female mice. (**C**) Splenocyte number from each group in male mice. (**D**) Spleen weight from each group in male mice. A one-way ANOVA with Bonferroni’s post-hoc test was performed to determine statistical significance. Combined data from 3 separate experiments (mean ± SEM) are shown. Each dot represents one mouse. ns: non significant *p < 0.05; ***p < 0.001.

**Figure 4:**
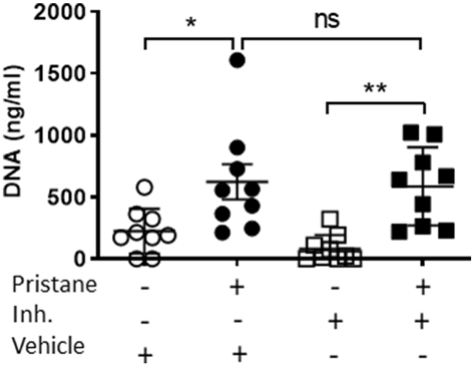
The benzimidazole inhibitor does not alter the pristane-induced cfDNA release in the serum of mice. DNA concentration (ng/ml) in the serum of female mice injected with pristane (0.5 ml) treated with PBS or the benzimidazole inhibitor twice per week for 8 weeks is shown. A one-way ANOVA with Bonferroni’s post-hoc test was performed to determine statistical significance. Combined data from 3 separate experiments (mean ± SEM) are shown. Each dot represents one mouse (n = 9 mice per group). *p < 0.05; **p < 0.01.

Having confirmed that the benzimidazole inhibitor is potent on classical TLR pathways, we i.p. injected C57BL/6J mice with 0.5 ml of pristane, a well-established model of murine sterile inflammation^14^. One week after pristane injection mice were treated with vehicle or benzimidazole inhibitor, twice per week for 8 weeks. Spleens were collected and splenomegaly was assessed by splenocyte number and spleen weight evaluation. We found that compared to control mice, pristane-injected females exhibited higher numbers of splenocytes than control mice, which were significantly reduced in pristane-injected mice treated with the inhibitor (Fig. 3A). Consistently, pristane-injected mice showed increased spleen weights, with a significant reduction when pristane-injected mice were treated with the inhibitor (Fig. 3B). Interestingly, whereas the inhibitor completely blocked the pristane-dependent increase in splenocyte numbers (Fig. 3A), it did not completely prevent the pristane-induced increase of spleen weight (Fig. 3B). Furthermore, we performed the same experiment and analysis in male mice, where we did not find any significant pristane-induced increase of either splenocyte number or spleen weight (Fig. 3C,D), suggesting that females are much more sensitive to pristane than males. For this reason, subsequent analysis from in vivo studies was performed only in females. Both male and female mice were still used for other in vitro assays. It is known that injected pristane induces nephritis in different mouse strains that models aspects of autoimmunity^36,37^. To evaluate renal inflammation in C57BL/6J mice, we carried out a mass spectrometry-based proteomics analysis that allowed us to identify possible variation of protein levels in each group. We identified three different proteins already shown to be putative markers of chronic renal inflammatory diseases in humans^38–41^: kininogen, kallikrein and fibronectin, that were significantly elevated in the urine of pristane-injected mice compared to controls. Consistent with our hypothesis, the inhibitor was able to block their pristane-associated increase (Table 1) compared to controls. Intriguingly, putative acute phase markers^42,43^ were not differentially expressed between the pristane group and the controls (Table 1), validating the pertinence of this model for studying chronic inflammation. However, we found that pristane could induce a significant increase of serum cfDNA compared to control mice and treatment with the benzimidazole inhibitor did not inhibit cfDNA release (Fig. 4). These results suggest that in females, the benzimidazole inhibitor reduces the pristane-dependent increase of splenic immune cell proliferation and infiltration and contributes to prevent other signs of inflammation (e.g. edema). Furthermore, our findings suggest that using the benzimidazole inhibitor may ameliorate pristane-associated chronic renal inflammation. Pristane may directly or indirectly induce cfDNA release, independently of the treatment with the benzimidazole inhibitor. This is important in our study because cfDNA is a potential TLR9 inducer and is used as a biomarker in a variety of human chronic inflammatory diseases, such as Systemic Lupus Erythematous (SLE) and Rheumatoid Arthritis (RA)^44^.

**Table 1:** Mass spectrometry data showing protein expression in the urine of different experimental groups.

Protein	PBS + vehicle (A)	Pristane + vehicle (B)	PBS + inhibitor (C)	Pristane + inhibitor (D)	*p* value B vs. A	*p* value D vs. C
**Chronic inflammation markers**						
Kininogen	1 ± 1.2	4.6 ± 2.3	2.6 ± 1.5	2.6 ± 2.2	0.01 (*)	0.99 (ns)
Kallikrein	8 ± 2.5	13.8 ± 3	10.8 ± 4.3	10.6 ± 3.4	0.01 (*)	0.94 (ns)
Fibronectin	0 ± 0	2.6 ± 2.2	0.8 ± 1.3	1.8 ± 2.2	0.03 (*)	0.40 (ns)
**Acute phase markers**						
Meprin A	7 ± 4.6	12.6 ± 7.5	10.4 ± 5.5	13.4 ± 7.5	0.19 (ns)	0.49 (ns)
Alpha-1-acid glycoprotein	0.75 ± 0.96	3 ± 2.58	0.5 ± 1	2.75 ± 2.22	0.15 (ns)	0.11 (ns)
**Not-regulated proteins**						
Lymphocyteantigen 6C1	1.4 ± 1.1	2.8 ± 2.4	1 ± 0.7	2.4 ± 2.3	0.27 (ns)	0.23 (ns)
Uromodulin	11.2 ± 2.8	13.6 ± 6.9	12.6 ± 3.8	15.8 ± 5	0.49 (ns)	0.29 (ns)
Protein AMBP	0.8 ± 1.1	3.2 ± 3.6	0.6 ± 0.9	3.2 ± 1.3	0.20 (ns)	0.11 (ns)

Combined data from 3 separate experiments are shown (n = 5 mice per group). Numbers represent spectral counts (mean ± SEM) of selected proteins for which Scaffold software identified 2 separate peptides. Refer to “Data availability” for more information on raw data.

### The benzimidazole inhibitor reduces the pristane-associated proinflammatory chemokine production

**Figure 5:**
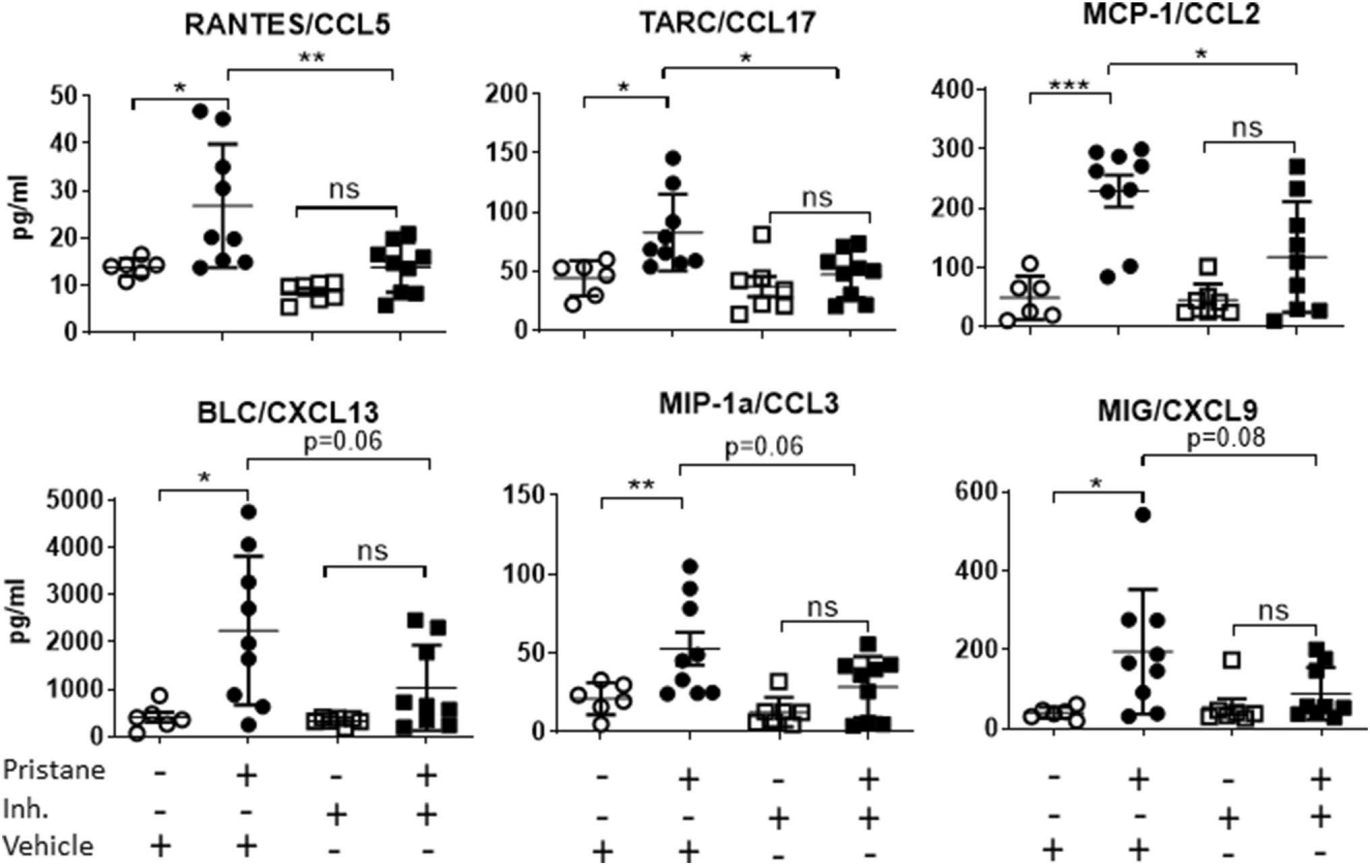
The benzimidazole inhibitor reduces proinflammatory chemokine production in sera of pristane-injected mice. WT female mice received a pristane injection (0.5 ml) and after one week were treated with the benzimidazole inhibitor twice per week for 8 weeks. Chemokine levels in sera were measured by a multiplex assay. (Upper panel) Chemokines significantly reduced by the benzimidazole inhibitor. (Lower panel) Chemokines slightly reduced by the benzimidazole inhibitor. A one-way ANOVA with Bonferroni’s post-hoc test was performed to determine statistical significance. Combined data from 3 separate experiments (mean ± SEM) are shown. Each dot represents one mouse (n = 6 to 9 mice per group). *p < 0.05; **p < 0.01; ***p < 0.001.

Next, we hypothesised that cfDNA release could trigger systemic inflammation; therefore we measured serum proinflammatory cytokines and chemokines. Release of these mediators is fundamental for immune cell recruitment and function, and an increase of their levels in serum has been associated with inflammatory disorders^45,46^. Even though we could not detect any pristane-dependent production of proinflammatory cytokines such as IL-6, IL-1β, IFN-β, TNF-α, IL-12(P70) and IFN-α (Supplementary Fig. S2), we measured 6 different chemokines that were significantly upregulated in the serum of pristane-injected mice (Fig. 5, 2 left groups). Intriguingly, the benzimidazole inhibitor was able to significantly (upper panel) or slightly (lower panel) reduce the pristane-associated chemokine secretion (Fig. 5). Most importantly, the benzimidazole inhibitor partially blocked increases in systemic chemokine levels compared to control mice (Fig. 5). In sum, these results suggest that the benzimidazole inhibitor could reduce the action of systemic cfDNA to induce chemokine production following pristane injection and possibly other inflammatory pristane-induced factors.

### Pristane activates macrophages independently of the benzimidazole inhibitor treatment

**Figure 6:**
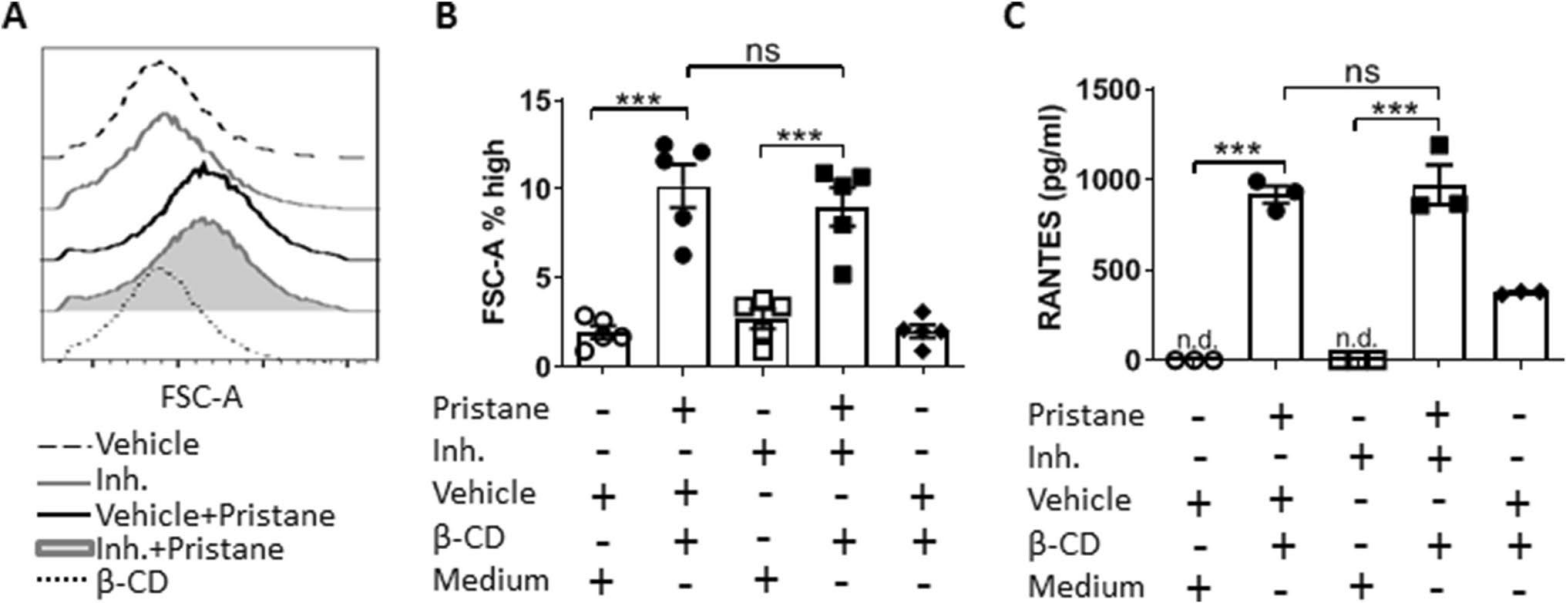
Pristane activates BMDMs independently of the benzimidazole inhibitor treatment. BMDMs pre-treated with either vehicle or the benzimidazole inhibitor (10 μM) for 30 min and stimulated with pristane/β-CD complexes for 48 h. (**A**) Cell size was evaluated measuring FSC-A by FACS. (**B**) Quantification of FSC-A showed in (**A**). (**C**) Secretion of RANTES detected in each group. In (**A**), a representative of five biological replicates is shown. In (**B**) and (**C**), a one-way ANOVA with Bonferroni’s post-hoc test was performed to determine statistical significance. (**B**) Shows combined data from 2 separate experiments with a total of 5 mice (mean ± SEM). (**C**) Shows combined data from one experiment with a total of 3 mice. Each dot represents one mouse (n = 3 to 5 mice per group). n.d., non-detected; **p < 0.01; ***p < 0.001.

Since the benzimidazole inhibitor successfully ameliorated pristane-dependent inflammatory disease manifestations in vivo, we postulated that pristane could induce inflammation through macrophage activation. To investigate this hypothesis, we assessed an in vitro experiment using pristane. To allow its miscibility in aqueous medium, pristane was included in complexes with β-Cyclodextrine (β-CD) as previously described^27^. BMDMs pretreated with the benzimidazole inhibitor or vehicle and subsequently stimulated with pristane (50 μM) appeared significantly more enlarged than control cells as per forward scatter measurement (Fig. 6A,B). To be sure that this phenotype was not due to the presence of β-CD, we also treated BMDMs with β-CD only, but no differences in cell size were found compared to controls (Fig. 6A,B). An increased cell size can be a sign of macrophage activation^47^; therefore, we measured proinflammatory mediator release in BMDMs and found that pristane-stimulated cells exhibited significantly higher RANTES levels, which were independent of the benzimidazole inhibitor pre-treatment (Fig. 6C). Of note, β-CD alone seemed to induce RANTES release as well, although it did so to a significantly lesser extent compared to pristane induction (Fig. 6C). Taken together, these data demonstrate that pristane does activate macrophages, although the mechanism by which this happens does not seem to be dependent of the benzimidazole inhibitor targets.

## Discussion

In recent years, several inhibitors of inflammatory pathways have been synthesised and tested in the context of multiple inflammatory disorders^8,9,48,49^. In this paper, we show that using a benzimidazole inhibitor ameliorates manifestations of sterile inflammatory disease by blocking the systemic production of proinflammatory chemokines. Our data demonstrate that this inhibitor might be used as a therapeutic in the treatment of sterile inflammatory diseases.

It is well known that a number of inflammatory disorders are TLR-dependent^2^. Therefore, we assessed the impact of the benzimidazole inhibitor on TLR-dependent responses in vitro. Treatment with the benzimidazole inhibitor significantly reduced TLR4- and TLR9-dependent IL-6 production and TLR9-dependent RANTES production in BMDMs, with the TLR9-dependent response being the most impaired by the inhibition (Fig. 1). This benzimidazole inhibitor has been shown to specifically inhibit two MyD88-dependent molecules, IRAK1 and IRAK4^19^. Upon TLR4 activation, TLR4 and its bound substrate are endocytosed, after which TLR4 signals through the TRIF-dependent pathway more than through MyD88-dependent signalling^50^. RANTES production is TRIF pathway-dependent (where IRAK1 and IRAK4 are not involved) rather than MyD88-dependent, which could serve as an explanation for the result observed in Fig. 1. Moreover, in our in vitro experiments, the benzimidazole inhibitor appeared to have other targets, since it reduced TLR3-dependent IL-6 and RANTES release (Fig. 1), which is IRAK1 and IRAK4 independent^3,51^. Consistent with this idea, a very recent study showed that IRAK4 shares 93% overall amino acid identity within the respective ATP binding pocket of TAK1, another kinase involved in inflammatory pathways; raising the possibility that some IRAK1/4 inhibitors (including the benzimidazole inhibitor) may have off-target effects through TAK1^52^. Since TAK1 has been shown to be involved in TLR3-dependent signalling^51^, a possible blockade of TAK1 by the benzimidazole inhibitor would explain the reduction of TLR3 signalling in vitro (Fig. 1). This additional unexpected inhibition of another TLR target broaden the scope of the benzimidazole inhibitor and its efficacy for sterile inflammation, especially when the TLR ligand is unknown. Alternatively, our result may be due to either some impurity in the TLR3 agonist (Poly I:C) or due to a potential paracrine/autocrine effect where TLR3 pathway-dependent mediators stimulate activation of the MyD88 pathway and MyD88-dependent molecules. In addition, we showed that the benzimidazole inhibitor could significantly downregulate molecular events downstream of TLR9 activation such as ERK-MAPK phosphorylation and NF-κB nuclear translocation (Fig. 2).

In the last decades pristane has been used to mimic environmentally-induced sterile inflammatory diseases, such as SLE and RA, in mice that are not genetically prone to these syndromes^10,14,53^. Although several papers have been published regarding adaptive immune responses towards pristane-induced Lupus^14,16–18^, there is still a large gap in our understanding of the role of the innate immune response. Furthermore, the actual mechanism by which pristane induces inflammation in vivo is largely unknown, except for some involvement of TLRs^54–56^. We found that the benzimidazole inhibitor is able to reduce the pristane-associated increase of splenic immune cell proliferation and infiltration, although it could not completely prevent other signs of inflammation, such as edema (Fig. 3). We did not see the same pattern in male mice, which turned out to be much less sensitive to pristane than females (Fig. 3C,D). To the best of our knowledge, for the first time a female sex bias has been shown in a pristane-induced inflammatory model in C57BL/6J mice. It is known how sex chromosome genes and sex hormones, including oestrogens, progesterone and androgens, contribute to the differential regulation of immune responses between the sexes^57,58^; thus, an interaction of pristane and/or the inhibitor with one or more of these sex-related factors might explain this interesting pattern. Given pristane’s ability to induce nephritis in different mouse strains^36,37^, our mass spectrometry-based proteomics analysis showed that the benzimidazole inhibitor might ameliorate chronic renal inflammation (Table 1). Next, we showed that pristane-associated symptoms may be driven by macrophages, which exhibited pristane-dependent enlargement (Fig. 6A,B) and RANTES release (Fig. 6C). Currently, it is unclear whether pristane is engulfed or simply bound to the cell membrane; however, the effect of the benzimidazole inhibitor did not modify macrophage size or RANTES levels, suggesting that the pristane-dependent macrophage activation process utilizes a pathway that is not targeted by the inhibitor. In vivo, we showed that pristane increases serum cfDNA levels (Fig. 4), although we could not detect any anti-dsDNA antibodies, which might be explained by the early time point (8 weeks post-pristane injection) used in this study (data not shown). Furthermore, cfDNA levels are independent of the inhibitor treatment (Fig. 4). Serum cfDNA increase is probably due to pristane-induced cell death, which is upstream of the benzimidazole inhibitor target. This result is consistent with data from others indicating that pristane might induce apoptosis in vitro and in vivo^59^. Interestingly, despite comparable serum cfDNA levels in control and treated mice, the benzimidazole inhibitor blocked serum proinflammatory chemokines such as MCP-1, RANTES and TARC (Fig. 5) showing its effect downstream of cfDNA stimulation. It is well known that under certain circumstances, self-derived DNA might be recognized by TLR9^60^; furthermore, TLR9 has already been shown to be required for the development of autoimmunity and Lupus nephritis in pristane-induced nephropathy^56^, with TLR9^−/−^ mice exhibiting predominant decrease in Th1 cytokine production, decreased anti-RNP antibody levels and renal injury^56^. The chemokines MCP-1, RANTES and TARC are indispensable for monocyte, macrophage and T cell chemoattraction at the site of inflammation, and our results are consistent with other published works reporting them to be upregulated in the serum of patients with active SLE^61^ and to be key molecules in the pathogenesis of many other sterile inflammatory diseases^62–64^. Nevertheless, the different outcomes obtained in regard to pristane-dependent RANTES release in vitro and in vivo (Figs. 5 and 6C) could be explained by the fact that BMDMs store this chemokine in the cytoplasm and pristane might induce its release. In vivo, it is possible that RANTES needs to be synthetized and perhaps TLR signalling is responsible for its synthesis after pristane stimulation. Of note, we did not detect any proinflammatory cytokine production 2 months following pristane injections (Supplementary Fig. S2), suggesting that in C57BL/6J mice pristane might chronically induce specific clusters of proinflammatory genes, rather than others. Sterile inflammatory responses are usually studied i.p. injecting pristane in Balb/c mice^14,37,65^. In this work, we demonstrated that pristane i.p. injected C57BL/6J female mice show signs of chronic sterile inflammation, being a valid addition to the Balb/c model.

Together our data demonstrate that using the benzimidazole inhibitor, exerting a major effect in inhibiting the TLR downstream effectors IRAK1 and IRAK4, results in the blockage of proinflammatory chemokines with a critical role in sterile inflammatory disease progression. Furthermore, treatment with the benzimidazole inhibitor might lead to an amelioration of some features of sterile autoinflammatory disease, such as renal dysfunction and splenomegaly. Collectively our findings demonstrate that the benzimidazole inhibitor might represent a new therapeutic in the recruitment of immune cells during environmentally-induced sterile autoinflammatory diseases in humans.

## Supplementary information

Supplementary information.

## Data Availability

The mass spectrometry proteomics data have been deposited to the ProteomeXchange Consortium via the PRIDE^66^ partner repository with the dataset identifier PXD017492. Reviewer account details: Username: reviewer72751@ebi.ac.uk, Password: ngM9wbls.
