# Supplementary material for: A benzimidazole inhibitor attenuates sterile inflammation induced in a model of systemic autoinflammation in female mice
Source: Sci Rep. 2020 Jul 21;10:12100. doi: 10.1038/s41598-020-68985-1 (PMC7374700; doi:10.1038/s41598-020-68985-1)
Supplement: Supplementary file 1 — Supplementary information. [file 41598_2020_68985_MOESM1_ESM.pdf]

A benzimidazole inhibitor attenuates sterile inflammation induced in a model of systemic autoinflammation in female mice

Federica Agliano[1], Keaton S. Karlinsey[1], Michael Ragazzi[1], Antoine Ménoret[1, 2,*], Anthony T. Vella[1,*]

[1]Department of Immunology, University of Connecticut Health Center, Farmington, CT, USA.

[2]Institute for Systems Genomics, UConn Health, Farmington, CT, USA.

*Corresponding author

Supplementary Figure S1

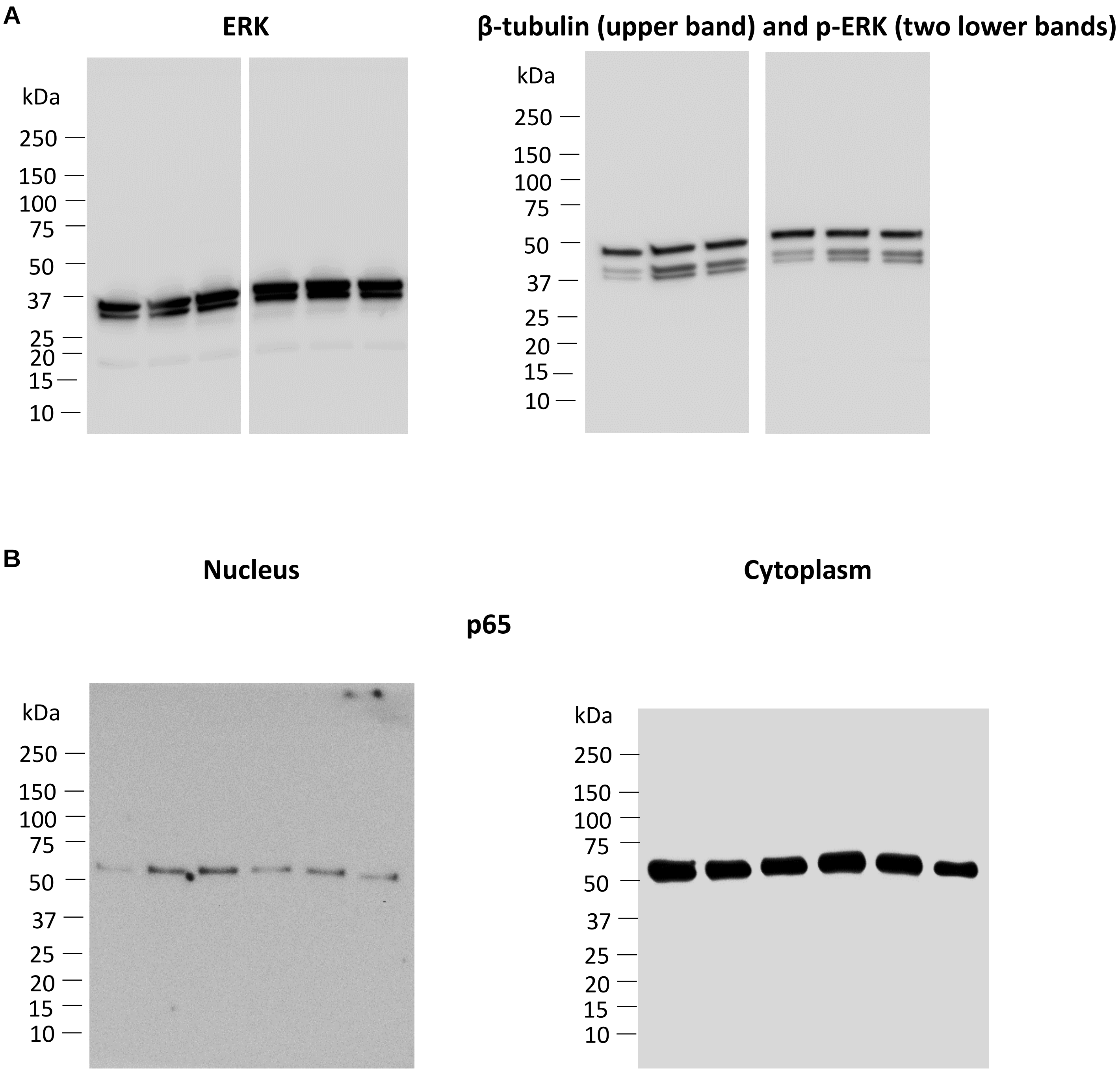

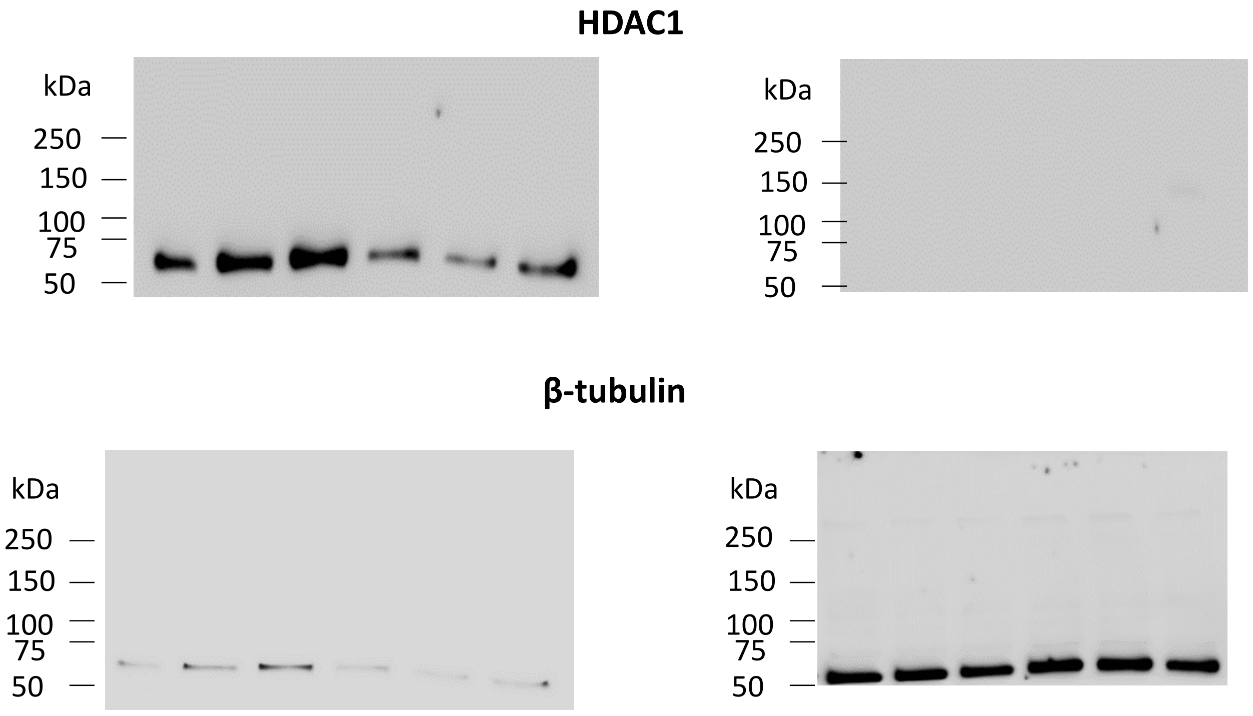

Supplementary Figure S1. A. Full-length membranes with bands showed in Fig. 2A. **B.** Full- or semi-length membranes with bands showed in Fig. 2C, E.

Supplementary Figure S2

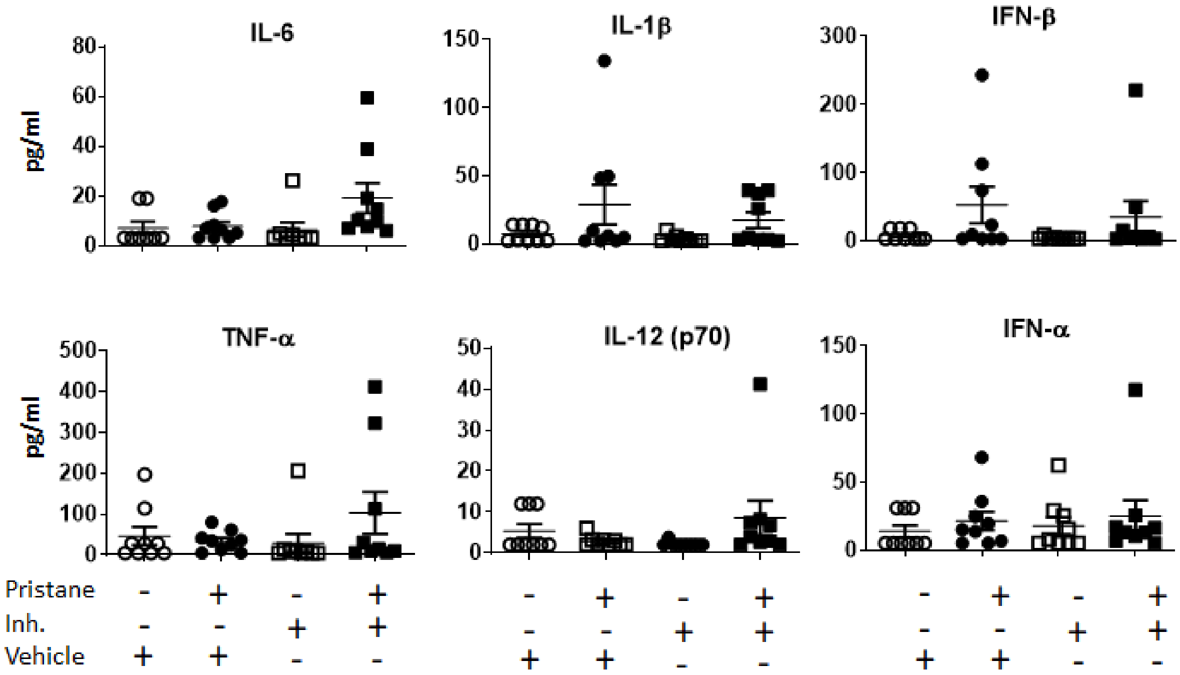

Supplementary Figure S2. The benzimidazole inhibitor does not reduce pro-inflammatory cytokine production in sera of pristane-injected mice. WT female mice received a pristane injection (0.5ml) and after one week were treated with the benzimidazole inhibitor twice per week for 8 weeks. Cytokine levels in sera were measured by a multiplex assay. A one-way ANOVA with Bonferroni post-test was performed to determine statistical significance. Combined data from 3 separate experiments (mean ± SEM) are shown (n=9 mice per group). Each dot represents one mouse.
